# Hospital and Institutional News

**Published:** 1918-03-02

**Authors:** 


### HOSPITAL AND INSTITUTIONAL NEWS.

### THE PRINCE OF WALES AND KING EDWARD VII.'s HOSPITAL.

WE are glad to record that at the last moment it became possible to include King Edward VII.'s Hospital, Cardiff, in the list of institutions which the Prince of Wales visited during his stay in South Wales last week. The chief hospital in his Principality, which was visited by the King and Queen in 1912, when they inspected the ward named to commemorate their Coronation, had therefore, one may submit, a personal as well as a public claim upon the Prince's attention. Indeed, we may recall that it contains a picture in tiles of his investiture at Carnarvon, which received the King's personal inspection, in consequence of which a detail was corrected in the final version of the work. We venture to think that on the present occasion it is worth while to emphasise these personal points, since the chief hospital of the Principality naturally cherishes these intimate links with His Royal Highness, knowing that on a visit to Wales like the present they did appeal to his sympathy as much as its public work, which is common knowledge. We therefore rejoice that the Prince's engagements allowed him to visit King Edward VII.'s Hospital, since nothing surely could have given a more graceful and appropriate finish to his recent tour.

### AN INTERESTING GUY'S LEGACY.

SECRETARIES of institutions do not often die rich, and, however much they are attached to the charities they have served, are not in a position to benefit them by will. Now and then, however, a hospital finds itself a legatee of a former officer. This is the case with Guy's Hospital, whose medical school has been left a substantial sum, running, we believe, into five figures, by Mr. C. H. Wells, for many years secretary to the treasurer. Mr. Wells, whose death was recently announced, left estate to the value of £72,347. After making provision for his wife and for the payment of a number of legacies and annuities, all the property passes to Guy's medical school. There are, no doubt, other instances of similar bequests, although not of similar size; the late chaplain of the National Hospital for the Paralysed and Epileptic left the hospital about £500, and we recollect that a late secretary of the Putney Royal Hospital for Incurables remembered several of his co-workers in his will. Another London hospital was bequeathed a handsome sum by one who had for many years acted as honorary secretary.

### SECRETARIES AND SALARIES.

THE board of management of Cheltenham General Hospital fixed February 25 as the last day to send applications for the secretaryship, which has been vacated by Mr. W. H. Head on his appointment as Mr. R. J. Coles' successor at Addenbrooke's Hospital, Cambridge. When we read the terms of the appointment we wonder how many applications were received; and, further, what was the standard of capacity attracted by them. Applicants, whose possession of previous hospital experience was insisted on, were offered a commencing salary of £200 a year. The hospital possesses 114 beds. According to recent returns its ordinary expenditure amounts to over £7,000 a year. It claims, of course, rank as a first-class institution. Under these circumstances can the salary be considered worthy of the responsibilities, likely to attract capable men, or one which shows that the board is aware of the standard that Cheltenham Hospital ought to set, and, consequently, to provide for? The £200 a year scale is the scale of an assistant secretary to a minor institution. It cannot command the ability required to administer an important hospital, and if, alas! a capable man might be found by stress of circumstances to accept such a post, it could never be to his interest to keep it. It is manifest that a hospital which does not attract the loyalty of men is one which only has itself to blame if those whose services it secures are either second-class men or merely birds of passage.

### PORTUGUESE PATIENTS AT EXETER.

IN consequence of a number of Portuguese having been employed in the Devonshire woods, the Board of Trade has arranged with the board of management of the Royal Devon and Exeter Hospital for their treatment at the hospital. The cost of treatment and maintenance has been provided by the authorities. One effect of this arrangement has been to make the patients' library inadequate, and there is now a demand not only for a few good Portuguese books, but also for French volumes for the benefit of the French-speaking Belgians who are still to be counted among the patients. There are two proposals in view which deserve attention. One is the recommendation that during the war and for six months after the wage limit for admission to the hospital should be raised from 25s. to 35s. a week. The other is a proposal to establish at Exeter a centre for the treatment of disabled soldiers similar to that already existing at Bristol. To provide this at Exeter a new building would be required. A site is available, and the scheme could only be carried out under the direct authority of the Government. If permission was received, and the cost of building provided by someone who wished to build a permanent memorial, a ground-floor ward would be built. This could be designed for later additions, and might become the nucleus of the nose, ear, and throat departments, for which additional accommodation is required. We are glad to note the many-sidedness of the Royal Devon Hospital's present activities. This evidence of public spirit is the best way to enlist sufficient local support to remove the present debt and increase the permanent income.

### THE ENGLISH COTTAGE HOSPITAL.

THE East Grinstead Cottage Hospital has lost a loyal officer by the retirement of the honorary secretary, Mr. H. A. Perkins. He has held the post continuously for thirty years, and his service proves the attraction to public-spirited men of one of the peculiar glories of English institutions—namely, our cottage hospitals. These have so favourably impressed Americans that we lately received a book urging their establishment in the United States, under the name of small community hospitals. The new honorary secretary is Mr. Miller.

### THE STAFF RECORD OF SUNDERLAND ROYAL INFIRMARY.

In lately giving the staff record of Halifax Royal Infirmary, we asked whether any other hospital could surpass the general length of service there disclosed. It is pleasant, therefore, to publish the record of long service achieved by the staff of the Royal Infirmary, Sunderland, which, to say the least, is no less remarkable. The secretary, Mr. Thomas Robinson, has held office for 35 years. Miss M. P. Thomson, who only retired five years ago, served for 39 years; her successor, Miss Amour, who was trained in the institution, has served it for 20 years. Among the sisters, the present night superintendent, Sister Carter, has served for 29 years, Sister Wallis for 21 years, and Sister Marr for 18 years, while Sister Anne has just retired on a pension after 30 years' service. Among the rest of the staff, the engineer, Mr. Thomas Blakey, has a record of 36 years' work; Mr. W. Potts, the chief porter, of 30 years; while Mr. R. Wilkinson, of the pathological and $x$-ray department, has served the hospital for 19 years, and Mr. J. Crawford, the warder, for 18. This is indeed a creditable record of service, and we leave our readers to decide whether it excels that of Halifax Royal Infirmary, unless another hospital can make an even better showing. If so, it is still open to dispute the pre-eminence of the Halifax and Sunderland Infirmaries.

### A TABLE OF WORKMEN'S SUBSCRIPTIONS.

By announcing a scheme for the organisation of workmen's subscriptions in connection with Blackburn Royal Infirmary, Mr. James Kay, the chairman of the hospital, gave a comparative list of workpeople's subscriptions in other towns of the North which will interest a wider audience. After pointing out that for twenty years the Blackburn workpeople's subscriptions had remained practically stationary, while a system of weekly contributions among the corporation's electricity employees at once had raised their particular yearly contribution from £4 to £51, Mr. Kay gave the following figures. By adopting the system of one penny a week subscriptions for their respective hospitals, Mr. Kay said, the Bolton workpeople, to a hospital of 138 beds, contributed yearly £5,604; Wigan, 156 beds, £8,059; Barrow-in-Furness, 60 beds, £4,634; Leicester, 294 beds, £17,500; Newcastle, 430 beds, £30,000; Chesterfield, 120 beds, £5,639; Coventry, 85 beds, £7,500; Stoke, 216 beds, £13,312. At Blackburn Royal Infirmary, where there are 152 beds, the workpeople's contribution is £2,873, or only £259 more than it was in 1897! Nobody contends that the number of workpeople in Blackburn has not increased during this interval, and the moral of these comparative figures, apart from their interest as a record, is that by the proposed scheme of organisation the contributions of the Blackburn workpeople could easily be doubled. If they were, the hospital would no longer have to rely, as it has done too long, on the generosity of a few benefactors who have again and again come to the rescue with exceedingly liberal gifts.

### A NEW "COUNTY HOSPITAL" AT BRIGHTON.

It has been decided, we gather, to re-christen the Brighton, Hove, and Sussex Throat and Ear Hospital by exchanging this somewhat cumbersome title for one which is more general without being less precise. The new name, the Sussex County Throat and Ear Hospital, is not only more euphonious and more dignified, but, so far as we can see, sacrifices nothing in directness and point. It is well to add, however, that no hospital can assume the rank of a county institution merely by changing its name. Therefore opportunity at once should be taken to reach the county standard by wiping out the deficit and leaving no stone unturned to improve the equipment, and to overcome the staff difficulties of the time. It is necessary, in short, to offer to the public some scheme of policy and development which will represent more than an appeal for funds, though to make that appeal at this moment is certainly opportune.

### WOMEN HOSPITAL SECRETARIES.

The tribute of thanks given to hospital secretaries by boards of management which appreciate the efforts required to maintain the hospital standard, a standard which demands both a high level of routine as well as driving force, have a special interest when paid to the relatively small class of women hospital secretaries. It is, therefore, pleasant to see that Miss Travis, secretary of Chelmsford Hospital, has earned the special thanks of the board for her share in the work during a busy and active period. When we recall that 618 civil patients were admitted during the past year, apart from the 533 cases received at the hospital hut, under the Essex branch of the Red Cross Society, it is clear that women secretaries, in these difficult days, have to tackle considerable administrative problems. One can but wonder how far women will create a permanent demand for their services in this capacity after the war.

### MONTGOMERY HOSPITAL'S JUBILEE.

On March 1 the Montgomery County Infirmary will have completed fifty years of work since the first day on which it was opened. By way of jubilee the board of management has decided to install electric power, not only for lighting but also for the new apparatus for $x$-ray and electrical massage, the former of which is being provided by the generosity of Major David Davies, the president of the hospital. His gift leaves a sum of £800 to be raised, which has already been in part subscribed by various private and public friends of the institution. The general public of the district served by the hospital is invited to provide the remainder, and the importance of the occasion is made clear by a souvenir pamphlet which accompanies the appeal. It is an inspiring record of quiet improvement and loyal service, in which a sketch of the hospital's history and a list of the principal officers are included. Since October 1914, when it was one of the first to be put upon the War Office list of auxiliary "A" hospitals, forty beds have been available for the wounded, at a sacrifice of about one-third of the beds maintained for civilians. On the anniversary the new $x$-ray installation will be exhibited, a public profession and a service of thanksgiving will be held, and in the afternoon Lieut.-Colonel J. Lynn Thomas will deliver an address on "Lessons in Surgery and Medicine from the War" at a meeting of the governors. These facts show what hospitals can do even under war conditions, and the board and Mr. E. C. Morgan are to be congratulated upon their plans for improving the occasion by introducing the general public to the hospital's work.

### PROGRESS AT ABERAVON.

ALTHOUGH the Aberavon, Port Talbot and District General Hospital has only been in existence three or four years, owing to the rapid development of the industries round about it the question of enlargement will have to be faced at the first suitable opportunity. This intimation was conveyed in a report presented by the secretary, Mr. W. R. Thomas, at the annual meeting on February 9. The past year has been one of considerable progress. At an early date the Rupert V. Hallowes, V.C., Memorial Ward will be formally handed over, and an $x$-ray apparatus is also to be installed. In spite of the fact that the cost per head, owing to the abnormal price of provisions, drugs, and surgical stores, worked out at £153 12s. 6d. last year, as against £80 14s. for the previous twelve months, 1917 closed with a credit balance of £569. This was due in a large measure to the high level reached by the subscriptions, practically all the works in the district contributing generously to the institution.

### FURTHER EXTENSION OF THE THIRD WESTERN GENERAL HOSPITAL.

EVER-INCREASING demands for beds for military patients are being made throughout South Wales, and authority after authority is giving up its suitable buildings for the purpose. The latest move in this direction on a large scale is at Bedwellty, where the Guardians are asked to surrender their workhouse and infirmary, which will provide accommodation for 500 beds in addition to the staff, for conversion into a hospital affiliated to the 3rd Western General at Cardiff. The Bedwellty Guardians are endeavouring to arrange for the distribution of the workhouse and infirmary inmates among other similar institutions belonging to neighbouring authorities.

### A CENSUS OF LIMBLESS CASES.

THE Earl of Plymouth, at the request of the executive committee of the Prince of Wales' Hospital, Cardiff, is making arrangements for the taking of a census of all the limbless cases in Glamorgan and the other Welsh counties. Lord Plymouth, in explaining the proposal to the Lord Mayor of Cardiff in a recent letter, stated that though the Prince of Wales' Hospital was primarily established for the fitting of artificial limbs in the cases of sailors and soldiers disabled during the war, when its resources are not fully absorbed by military needs its facilities will be at the disposal of civilians. A census is therefore being undertaken in order that reliable data may be obtained as to the probable extent of the demand which civilians may make upon the hospital's services.

### A MUSEUM'S WAR RECORD.

THE share of science in the war is a topic that has become monotonous by overmuch repetition, but a recently published statement of the war services of the staff of the Natural History Museum refutes in a striking manner the allegations that young men of military age are finding safe shelter in Government departments. Sixty-one members of the staff are serving with the naval and military forces, twelve have joined the Volunteers, and six are serving as special constables and as orderlies in a London ambulance column. Of the pre-war staff there is not left one man of military age passed fit for general service. The museum has, moreover, been able to render signal assistance to various Government departments in problems arising from the war. Such have been the investigation of the wood used in the construction of a Zeppelin propeller brought down in this country, of a practical method of destroying the bugs that infest certain types of military bedding, and of mites that destroy oats given to horses at the Front. In other matters where the advice of the museum authorities has been sought, there have been questions as diverse as the advisability of introducing reindeer into South Georgia, and of discovering the particular genus of crustacean supplied by a certain firm as tinned lobster. Who nowadays will be bold enough to say that the man of science is remote from the interests of his fellow-men.

### "KITCHENER HOUSE."

THE time of a wounded man must often hang heavily upon his hands, and it would be well if voluntary or Government funds would provide for his service many institutions on the model of Kitchener House in Cambridge Gate, where the completion of a year's work was signalised last week by an informal gathering and discussion. Its aims, briefly, are to provide instruction, coupled with rest and recreation, for men in that transition stage of being still in hospital but allowed out during stated periods to amuse themselves as best they can. Classes are held in French and other languages, shorthand, typewriting and bookkeeping, picture-frame making, bookbinding, and metal work. In addition, a comfortable recreation room, with books and papers, a piano and gramophone, easy-chairs, and all the amenities of a well-appointed club, establishes an atmosphere of friendliness calculated to intrigue the most retiring. Last year the club was patronised by nearly 6,000

soldiers, of whom some forty per cent. availed themselves of its educational facilities. There is reason to believe that a larger proportion would be ready to do so if arrangements could be made for their regular transport to and from the hospital. As far as is possible this is already being done by the club authorities, but if the good offices of the Pensions' Ministry could be enlisted that would be very welcome.

### "THE BATH BUN."

The book of the Bath War Hospital, which was lately published under the title of "The Bath Bun," is a remarkable production. After seven weeks had passed since the idea was first discussed, the book, of 120 odd pages, was compiled and illustrated without any help, we understand, from outside contributors. For the illustrations the editor mainly relied on Sergeant J. K. Waring, who designed the cover and a mass of drawings in the text. He has never, we learn, received any drawing lessons, and this is presumably the first important field that his genuine talent has found. The twelve hundred patients at Combe Park supported their editor, Private C. Hampton Thorp, so well that we hope "The Bath Bun" may develop at least into a quarterly. It aims, on the whole, at a high standard, and the editor has in his drawer a series of French landscapes in water-colours by Lance-Corporal Madigan which we should be glad to see in reproductions. After humorous sketches of various kinds we turned with interest to the article on Rapello by the chaplain, the Rev. D. J. Pring, to Mr. J. M. H. Monro's "Impressions of an Anglo-Belgian Hospital," and to Lieut.-Colonel Egbert Lewis' account of "A Visit to the Western Front." There are some good parodies. Perhaps the best is the epigram entitled "To any Sister"; "Hiawatha's" parody of Henley's unrhymed verses on hospital life. We must also mention Herbert Lambert's photographic study of a Boy Scout, so different in sentiment from the vivid pen-and-ink sketches of Sergeant Waring which make many a page reminiscent of *Punch*, where, perhaps, his work may yet be seen and appreciated. There is also promise in Miss Stuart Clerk's verses entitled "Bed-Time." If her touch is a trifle sentimental, she has an ear for rhythm and a hint of that restraint which is the beginning of style. In short, "The Bath Bun" should appear as regularly as its namesake, since the ingredients for the gazette are evidently abundant.

### THE "SOUTHERN CROSS" IN ECLIPSE.

Two changes in the *Southern Cross*, the gazette of the 1st Southern General Hospital, Birmingham, have occurred together: the retirement of the editor, and the appearance of the paper every other month only. The latter change is more truly an eclipse, we hope, than the former, for a good editor, among his many duties, always trains another to follow him. It is amusing to those who, from the impartial point of view of the critic, have had to complain of the formality of most inspections, to read the article in this gazette alleging the panic created when the staff learns that an inspection is about to take place. A real humorist would have found his fun in the contrast between the panic of expectancy and the, more or less, tameness of the occasion. The drawings and verses do not call for special comment, but when the new editor gets into his, or her, stride, the larger interval between each issue caused by the publication of the gazette in each alternate month should enable him to present a varied selection. If the next issue is published up to time it will appear in March, and we shall look forward to its publication.

### CHURCH COLLECTIONS FOR HOSPITAL SUNDAY.

While hospitals all over the country are, of course, grateful for the financial support received in the way of church and chapel collections, there is one point which gives cause for anxiety in a number of towns. A tendency has sprung up recently for churches to take the annual collection on some day other than the appointed Hospital Sunday, and the invariable result of this lack of unity is a decrease in the gross proceeds from this source of revenue. Hospital boards of management will find it to their interest to leave no stone unturned to secure unanimity in the appeal from the pulpits on behalf of their institutions. Otherwise the interest will be weaker, and there will inevitably follow a decline in the financial aid which Hospital Sunday affords.

### THIS WEEK'S DRUG MARKET.

As was not unexpected, higher prices are now asked for bromides, and in view of the falling-off of supplies from the United States it is not improbable that a further advance in quotations will take place. Makers of strychnine have increased their prices by 20 per cent.; this is one of a series of advances due to the scarcity of the raw material—nux vomica. Lithia carbonate and lithia citrate are dearer. Balsam Tolu continues very scarce and dear. The scarcity of sugar of milk has become very acute, and even at famine prices it would be difficult to buy in any considerable quantity. Cream of tartar is again dearer. The possibility of a maximum price being fixed for saccharin is being discussed; this sweetening agent having become popular as an article of diet, and a regular adjunct to the tea-shop, it would not be surprising if the Food Controller took some steps to ensure a proper distribution of the limited supplies available. The difficulty is that at present we are dependent upon the United States of America for the bulk of our supplies, and that the exports from that country may become much smaller. Moreover, the price in New York has recently fluctuated a great deal, and although a figure based on the present selling price in New York would come very much below the price at which saccharin is being sold here, it should not be forgotten that the bulk of the stocks in this country was bought in New York when the price was substantially higher than it is at present. It is clearly desirable that dealers should be content with reasonable profits, but the difficulties in the way of fixing a maximum price are considerable.

